# IMPACT OF SLEEVE GASTRECTOMY ON THE NEUTROPHIL-TO-LYMPHOCYTE RATIO AND THE PLATELET-TO-LYMPHOCYTE RATIO AND ITS RELATIONSHIP WITH POSTOPERATIVE WEIGHT LOSS

**DOI:** 10.1590/0102-67202025000013e1882

**Published:** 2025-05-23

**Authors:** Tiago Cavalcanti IWANAGA, Fernando SANTA-CRUZ, Álvaro Antonio Bandeira FERRAZ, Flávio KREIMER

**Affiliations:** 1 Universidade Federal de Pernambuco, Postgraduate in Surgery - Recife (PE), Brazil;; 2 Universidade Federal de Pernambuco, Department of Surgery - Recife (PE), Brazil.

**Keywords:** Bariatric Surgery, Inflammation, Obesity, Inflammation Mediators, Cirurgia Bariátrica, Inflamação, Obesidade, Mediadores da Inflamação

## Abstract

**BACKGROUND:**

Obesity represents a chronic pro-inflammatory status that contributes to accelerated atherosclerosis and cell aging. Besides the widely used C-reactive protein and ferritin, other inflammatory markers have gained attention, such as neutrophil-to-lymphocyte ratio (NLR) and platelet-to-lymphocyte ratio (PLR), which are related with the degree of inflammation in various pathological conditions, including obesity and its comorbidities.

**AIMS:**

To compare and monitor the levels of NLR and PLR before and after sleeve gastrectomy (SG).

**METHODS:**

Retrospective study that included a total of 622 patients with obesity who underwent SG as primer bariatric surgery in our center. Data regarding the presence of comorbidities, including type 2 diabetes (T2D), high blood pressure (HBP) and non-alcoholic fatty liver disease (NAFLD), variations in body weight and body mass index (BMI), and biochemical markers of inflammation, including neutrophil-to-lymphocyte ratio (NLR), platelet-to-lymphocyte ratio (PLR) and C-reactive protein (CRP) were gathered. Values of NLR and PLR were correlated with weight loss and prognosis of comorbidities within the postoperative period.

**RESULTS:**

The sample was predominantly female (79.3%) with average age 36.91±10.04 years, with comorbidities including HBP (25.1%), T2D (8.0%), and NAFLD (80.1%). Patients with HBP showed reduced NLR and CRP post-intervention, while those with T2D experienced decreased CRP but increased PLR. Correlation analysis found no significant correlation between BMI/weight changes and NLR but significant correlation with PLR. Post-surgery, NLR decreased for previously NAFLD patients, and PLR increased.

**CONCLUSIONS:**

According to the results, patients with obesity present a significant decrease in NLR and an increase in PLR after SG.

## RESUMO

**RACIONAL:**

A obesidade representa um estado pró-inflamatório crônico que acelera a aterosclerose e o envelhecimento celular. Além da proteína C-reativa (PCR) e da ferritina, outros marcadores têm ganhado atenção, como a razão neutrófilos/linfócitos (RNL) e a razão plaquetas/linfócitos (RPL), que são relacionados com o grau de inflamação sistêmica em várias patologias, incluindo a obesidade e suas comorbidades.

**OBJETIVOS:**

Comparar os valores de RNL e RPL antes e depois da gastrectomia vertical (GV).

**MÉTODOS:**

Estudo retrospectivo que incluiu 622 pacientes portadores de obesidade submetidos à GV em nosso centro. Dados relacionados à presença de comorbidades, incluindo diabetes tipo 2 (DT2), hipertensão arterial sistêmica (HAS) e doença hepática gordurosa não alcoólica (DHGNA), variações de peso e índice de massa corporal (IMC) e marcadores bioquímicos de inflamação sistêmica, incluindo RNL, RPL e PCR, foram coletados. Os valores da RNL e RPL foram correlacionados com a perda de peso e o prognóstico das comorbidades no período pós-operatório.

**RESULTADOS:**

A amostra foi predominantemente feminina (79,3%), com idade média de 36.91±10.04 anos, com a presença das seguintes comorbidades: HAS (25,1%), DT2 (8,0%), e DHGNA (80,1%). Pacientes com HAS apresentaram redução nos valores de RNL e PCR após o procedimento, enquanto os pacientes com DT2 apresentaram redução de PCR e aumento da RPL. Não houve correlação das variações do IMC com a RNL, porém houve correlação significativa com a RPL. Após a cirurgia, a RNL reduziu e a RPL aumentou nos pacientes com DHGNA.

**CONCLUSÕES:**

Pacientes com obesidade apresentam redução significativa da RNL e aumento da RPL após a realização da GV.

**Central Message**

Obesity is also associated with a chronic state of low-grade inflammation, which plays a key role in the development of a variety of diseases and occurs mainly in the adipose tissue, where immune system cells, such as macrophages, lymphocytes and neutrophils, are present in high quantities. The ratios between neutrophils/lymphocytes (NLR) and platelets/lymphocytes (PLR) are conceived as markers to assess the degree of body inflammation resulting from various pathologies, including obesity. It is plausible to study the impact of the surgical treatment for obesity on the inflammatory status of patients through the assessment of NLR and PLR, trying to correlate its evolution with the postoperative weight loss.

**Perspectives**

Patients with obesity present a significant decrease in NLR and an increase in PLR after sleeve gastrectomy. These markers can interfere with the evolution of high blood pressure, type 2 diabetes and non-alcoholic fatty liver disease, in the postoperative period. Furthermore, the greater the difference in PLR values before and after surgery, the smaller the variation in body mass index (BMI).

## INTRODUCTION

Obesity is a condition characterized by the excessive accumulation of adipose tissue in the body^6^. It is an important risk factor for a variety of chronic diseases, including cardiovascular diseases, type 2 diabetes (T2D), certain types of cancer, liver disease, and respiratory disorders^2^ ^,^ ^8^. In addition to accumulation of fat, obesity is also associated with a chronic state of low-grade inflammation^22^. This inflammation plays a key role in the development of a variety of diseases and occurs mainly in the adipose tissue, where immune system cells, such as macrophages, lymphocytes and neutrophils, are present in high quantities^1^ ^,^ ^2^ ^,^ ^12^ ^,^ ^18^. This pro-inflammatory state and cell imbalance observed in patients with obesity is the consequence of an increased production of adipokines, such as tumor necrosis factor alpha (TNF-a), interleukin-6 (IL-6), and C-reactive protein (CRP), by the adipocytes^17^.

NLR and PLR are conceived as markers to assess the degree of body inflammation resulting from various pathologies, including obesity^9^ ^,^ ^14^. Taking this scenario into consideration, it is plausible to study the impact of the surgical treatment for obesity on the inflammatory status of patients through the assessment of NLR and PLR, trying to correlate its evolution with the postoperative weight loss. The objective of this study is to compare and monitor the levels of the aforementioned inflammatory markers before and after sleeve gastrectomy (SG).

## METHODS

### Study design

This is a retrospective cohort study analyzing all patients undergoing SG in our center between January 2012 and December 2022. It conducts a comparative analysis of PLR and NLR values pre- and post-surgery.

All procedures performed in this study involving human participants were in accordance with the ethical standards of the institutional research committee, the 1964 Helsinki declaration and its later amendments. The Ethics Committee of the Institution approved this research’s protocol (number 7.1223523.9.0000.5208).

### Study population

The sample was non-probabilistic, selected by convenience, totaling 1,499 patients. The sample included patients of both genders, aged between 16 and 65 years, who underwent SG at our center within the study period above and who had a postoperative follow-up period longer than one year, with results of the blood tests in both pre- and postoperative periods. Patients with incomplete medical records and those who presented postoperative complications, such as fistulas and bleeding, were excluded from the analysis. Patients who underwent SG as revisional surgery were also excluded.

### Data collection

Data were collected from an electronic medical record database. The demographic and anthropometric data of the studied patients were recorded, in addition to comorbidities, including hypertension, T2D, dyslipidemia, and non-alcoholic fatty liver disease (NAFLD). Concerning the quantitative variables, the values of blood count parameters, fasting blood glucose, total cholesterol and fractions, and C-reactive protein were recorded. PLR and NLR were calculated. All variables were studied at two different stages: before surgery and at least one year after surgery.

NAFLD was defined by the presence of significant hepatic steatosis (by imaging or histology) and the absence of other causes of chronic liver disease and steatosis, including significant alcohol consumption^5^ ^,^ ^13^. Hepatic steatosis was determined in the preoperative workout through an abdominal ultrasound examination and was graded - grade 1: liver echogenicity increased; grade 2: hyperechogenic liver obscuring the walls of the portal venous branches; and grade 3: hyperechogenic liver obscuring the diaphragmatic outline^16^.

### Statistical analysis

This study builds a database using a Microsoft Excel spreadsheet, exported to the Statistical Package for the Social Sciences (SPSS) software, version 21, which conducted the data analysis. To characterize the patients’ personal and clinical profile, a frequency distribution was produced, and the χ^2^ test was applied to compare ratios. To assess the normality of biochemical markers, the Shapiro-Wilk test was applied. If there was data normality, mean and standard deviation were calculated. Student t-tests for paired samples compared marker means before and after surgery. Also, a comparative analysis of the effects of surgery was carried out according to the presence or not of comorbidity. Furthermore, the correlation between the parameters NLR and PLR with the levels of glucose, C-reactive protein (CRP), high-density lipoproteins (HDL), low-density lipoprotein (LDL) and very-low-density lipoprotein (VLDL) was evaluated before and after surgery using the Pearson correlation test. All results complied to a significance level of 5%.

## RESULTS

The present study included, in its final analysis, 622 patients with obesity who underwent SG. The sample comprised mainly female individuals (79.3% x 20.7%), with an average age of 36.91±10.04 years. Regarding comorbidities, 25.1% of the sample had hypertension, 8.0% had T2D and 80.1% NAFLD (Table 1).

**Table 1:** Demographic profile of the sample.

Variable	n	%	p-value
Gender			
Male	129	20.7	<0.001
Female	493	79.3	
Age (Mean±SD)	36.91±10.04	-	
Comorbidity			
HBP	156	25.1	<0.001*
T2D	50	8.0	
Hepatic steatosis			
Grade 1	194	31.2	<0.001*
Grade 2	286	46.0	
Grade 3	18	2.9

*p-value of χ^2^ test for ratio comparison.

HBP: high blood pressure; T2D: type 2 diabetes; SD: standard deviation.

Table 2 shows a comparison of biochemical and anthropometric variables according to the moment of evaluation. There was a significant reduction in all variables evaluated, except for PLR, which showed an increasing trend.

**Table 2:** Comparison among biochemical markers according to assessment group and moment of intervention.

Variable	Moment of evaluation		
	Pre-operative	Postoperative	p-value
NLR	2.36±0.95	2.09±0.84	<0.001
PLR	134.95±41.65	138.24±44.61	0.049
Weight	108.89±16.84	81.39±14.98	<0.001*
BMI	39.92±4.58	29.85±4.47	<0.001*
Glucose	99.36±24.77	86.59±16.04	<0.001*
CRP	3.65±5.82	2.46±5.01	<0.001*
Total cholesterol	197.98±37.92	185.33±36.43	<0.001*
HDL	48.27±12.24	46.59±10.86	<0.001*
LDL	122.11±31.25	118.40±32.04	0.002*
VLDL	27.61±14.05	20.27±8.83	<0.001*

*p-value of Student t-test for paired samples.

NLR: neutrophil-to-lymphocyte ratio; PLR: platelet-to-lymphocyte ratio; BMI: body mass index; CRP: C-reactive protein; HDL: high-density lipoproteins; LDL: low-density lipoprotein; VLDL: very-low-density lipoprotein.

Table 3 shows the analysis of inflammatory markers (NLR, PLR and CRP) according to preoperative comorbidities. After the intervention, for patients who had HBP, there was a significant reduction in NLR and CRP. For patients who had T2D, there was a significant decrease in CRP and an increase in PLR after the test.

**Table 3:** Analysis of markers according to preoperative comorbidity.

Variable	Comorbidity		
	HBP	T2D	NAFLD
NLR			
Before	2.36±0.89	2.09±0.74	2.34±0.93
After	2.06±0.79	2.27±1.25	2.08±0.81
p-value	<0.001*	0.290*	<0.001
PLR			
Before	129.12±37.33	129.81±36.17	133.01±41.23
After	132.16±40.24	149.94±63.47	137.90±41.69
p-value	0.320*	0.015*	0.005
CRP			
Before	3.16±4.97	5.73±8.97	3.55±5.46
After	2.09±4.28	3.61±5.41	2.35±4.94
p-value	0.002*	0.032*	<0.001

*p-value of Student t-test for paired samples.

HBP: high blood pressure; T2D: type 2 diabetes; NAFLD: non-alcoholic fatty liver disease; NLR: neutrophil-to-lymphocyte ratio; PLR: platelet-to-lymphocyte ratio; CRP: C-reactive protein.

Table 4 shows a correlation analysis of NLR and PLR variations before and after intervention with weight loss and BMI reduction. The difference in BMI and weight before and after surgery did not show a significant correlation with differences in NLR. However, there was a significant correlation between PLR and differences in BMI and absolute weight between pre- and postoperative periods (p=0.042, r=-0.082; p=0.016, r=-0.097, respectively).

**Table 4:** Correlation analysis of neutrophil-to-lymphocyte and platelet-to-lymphocyte ratios with variations in body mass index, absolute weight, and plasma glucose after sleeve gastrectomy.

Variable	NLR variation		PLR variation	
	Correlation	p-value*	Correlation	p-value*
BMI variation	-0.039	0.336	-0.082	0.042
Absolute weight variation	-0.041	0.306	-0.097	0.016
Plasma Glucose	-0,035	0,394	-0,110	0,007

*p-value of correlation test and Pearson.

NLR: neutrophil-to-lymphocyte ratio; PLR: platelet-to-lymphocyte ratio; BMI: body mass index.

Table 5 shows that there was a significant decrease in the NLR value after surgery for the group of patients who had NAFLD before the intervention (p<0.001) and a significant increase in the PLR value (p=0.005) before and after surgery. Patients who did not have NAFLD at baseline presented a significant decrease in the mean NLR (p=0.001). Regarding PLR, there was no significant change between pre- and post-intervention periods (p=0.501).

**Table 5:** Mean and standard deviation of increase/decrease in neutrophil-to-lymphocyte and platelet-to-lymphocyte ratios by group of patients according to the presence or not of non-alcoholic fatty liver disease.

Evaluated factor	NAFLD	
	Yes	No
NLR		
Before	2.34±0.93	2.47±1.02
After	2.08±0.81	2.11±0.97
p-value	<0.001*	0.001*
PLR		
Before	133.01±41.23	142.74±42.57
After	137.88±41.69	136.61±54.94
p-value	0.005*	0.501*

*p-value of Student t-test for paired samples.

NAFLD: non-alcoholic fatty liver disease; NLR: neutrophil-to-lymphocyte ratio; PLR: platelet-to-lymphocyte ratio.

## DISCUSSION

This study aimed to compare and monitor the levels of the inflammatory markers NRL and PLR before and after SG in patients with obesity and correlate their values with weight loss and the presence of comorbidities such as HBP, T2D and NAFLD.

Kashihara et al. conducted a retrospective study with a small sample (n=15) and studied the relationship between NLR and excess weight loss in patients who underwent SG within one year of follow-up. The authors identified that low NLR values (2.03) during the preoperative period are related to greater weight loss in the postoperative period (% excess weight loss - %EWL>50%)^11^. Furthermore, Zubiaga and Ruiz-Tovar also evaluated the potential prognosis of NLR and PLR for weight loss after SG, observing a negative correlation between NLR and %EWL (Spearman 2.525), and no correlation between PLR and %EWL or BMI during the postoperative period^24^.

The NLR has also been implicated as a potential predictive marker for early complications in the postoperative period of elective and emergency surgeries^3^ ^,^ ^7^ ^,^ ^21^. Da Silva et al. observed, in a retrospective analysis, that a NLR=10 in the first postoperative day of bariatric surgery is significantly associated with major complications (odds ratio - OR=3.71; 95% confidence interval - 95%CI 1.76-7.82), reoperation (OR=3.63, 95%CI 1.14-11.6) and prolonged postoperative length of stay (OR=3.70, 95%CI 2.2-6.22)^4^.

In contrast to the studies above, there was no correlation between the variation in NLR and weight loss in the present study. However, the current analysis did identify a statistically significant correlation (p=0.042 and 0.016) between the variation in PLR values during the pre- and postoperative periods and decreases in BMI and absolute weight. These findings allow stating that the greater the variation in PLR, the greater the weight loss, and consequently the decrease in BMI after surgery.

Kashihara et al. also reported in their cohort that lower NLR values are related to an improvement in T2D one year after surgery, occurring complete remission in 40%, partial remission in 20% and an overall improvement in 40% of cases^11^. Moreover, Zubiaga and Ruiz-Tovar observed a correlation between preoperative NLR values and plasma glucose (Spearman 0.685; p=0.02, p<0.05) and homeostatic model assessment for insulin resistance (HOMA-IR) (Spearman 0.764; p=0.01, p<0.05), in addition to an association between lower NLR values and T2D remission five years after surgery^24^.

In the present study, which compared blood glucose values with the markers NLR and PLR, there was a significant correlation between PLR and glucose levels (p=0.007, p<0.05), which allows stating that, for a decrease of one unit in glycemia, there is an increase by 0.110 points in the PLR ratio. In general, there is an increase by 3.29 points in the PLR ratio before and after surgery (p=0.049, p<0.05).

Among the patients included in this study, 116 patients had a preoperative diagnosis of T2D. Cross-checking the data and correlating patients with T2D and levels of NLR and PLR, there was a significant correlation between T2D and increases in PLR levels after sleeve SG (p=0.049, p<0.05) and no correlation between NLR levels. This result differs from that found by Zubiaga and Ruiz-Tovar^24^ and Kashihara et al.^11^, who reported significant correlation between T2D and NLR levels.

Still in the present study, the correlation between patients with HBP and levels of NLR and PLR was shown to be significant only for the decrease in the NLR. On the other hand, there was no statistically significant relationship with PLR levels. Overall, the results suggest a limited role of PLR count as a prognosis marker for HBP after SG.

Uehara et al. showed that both SG and Roux-en-Y gastric bypass (RYGB) promote a reduction of NAFLD due to the consequent weight loss and the metabolic aspects proper to the procedure, especially the action of incretins such as glucagon-like peptide-1 (GLP-1) and glucagon-like peptide-2 (GLP-2)^20^. Shavakhi et al. carried out a systematic review including 13 studies. The review showed that patients with nonalcoholic steatohepatitis (NASH) had high levels of NLR compared to patients without NASH (mean standard deviation - MSD=0.97, 95%CI 0.59-1.39, p<0.001)^19^. The sensitivity and specificity of NLR was 78.16% (95%CI 73.70-82.04%) and 76.93% (95%CI 70.22-82.50%), respectively. The conclusion is that the NLR ratio is a promising biomarker that can be integrated into clinical practice for predicting and preventing NASH in patients with NAFLD^19^. Zhou et al. reported that the relationship between NLR and PLR with NAFLD was not linear after adjusting for confounding factors. Their results suggested that a PLR=42.29 may be a protective factor for NAFLD, while a NLR <1.23 could be a risk factor for NAFLD^23^.

The present study showed that the vast majority of patients who underwent SG had NAFLD (80.1%). A moderate degree of steatosis (NAFLD) is the most common finding, which was present in 46% of the included patients. The literature is scarce, and the research conducted here did not identify other studies that showed a relationship between the values of NLR and PLR and an improvement in NAFLD after SG.

There was a significant decrease in the NLR (p<0.001) and increase in the PLR value (p=0.005, p<0.05) after surgery for the group of patients who had NAFLD before the intervention. Patients who did not have NAFLD at baseline presented a significant decrease in the mean NLR (p=0.001, p<0.05). Regarding PLR, there was no significant change between the pre- and post-intervention periods (p=0.501, p>0.05) for this group. This allows stating that patients with a previous diagnosis of NAFLD tend to present a significant decrease in NLR and increase in PLR after bariatric surgery, and this could be the cause or consequence of the overall improvement in the chronic inflammatory state induced by obesity.

McOwan et al. evaluated the relation between NLR and CRP in patients who underwent SG. As expected, based on the previous literature reviewed here, they found significant reductions in both NLR (pre: 2.4±1.59 *vs*. post: 1.7±0.86; p<0.001) and CRP (pre: 5.6±3.17 *vs*. post: 2.1±2.35 mg/L; p<0.001)^15^.

Jamialahmadi et al. conducted a prospective study with 90 patients and reported a relation between RYGB, weight loss, NAFLD and CRP^10^. The conclusion was that bariatric surgery led to a significant decrease in CRP values and hepatic steatosis and that CRP values before surgery cannot predict the success of weight loss and the liver status after RYGB^22^.

The current study showed that SG was responsible for a significant decrease in CRP values (3.65-2.46, p<0.001) and that this decrease also occurs in patients who present HBP and T2D. This shows that the improvement in systemic inflammation occurs regardless of these comorbidities. Correlating CRP values with the NLR and PLR shows that the decrease in CRP is correlated with the decrease in NLR values, but not correlated with PLR levels.

This study has some limitations that should be mentioned. Firstly, it is worth mentioning the gaps in the records of exams that could otherwise be extremely valuable in monitoring the outcome of some pathologies that this study aimed to evaluate, including the absence of triglyceride levels, the non-use of elastography in monitoring the degree of liver fibrosis, and the difficulty in finding and defining which diabetic patients presented complete remission, partial remission or improvement only. Also, this study was limited to a short follow-up time and consisted of an observational and retrospective analysis.

## CONCLUSIONS

According to the results, patients with obesity present a significant decrease in NLR and an increase in PLR after SG. These markers can interfere with the evolution of HBP, T2D and NAFLD in the postoperative period. Furthermore, the greater the difference in PLR values before and after surgery, the smaller the variation in BMI. Further studies, especially controlled and randomized trials, are needed in order to evaluate patients prospectively and determine the true relation between NLR and PLR and the remission of comorbidities, weight loss, and surgical complications.
